# Therapeutic efficacy of nimodipine and topiramate on migraine and vestibular migraine; A prospective multicenter open-label study

**DOI:** 10.1371/journal.pone.0344948

**Published:** 2026-03-19

**Authors:** Seo-Young Choi, Sun-Young Oh, Hyun Ah Kim, Ji‑Yun Park, Jae-Hwan Choi, Eun Hye Oh, Jeong-Yoon Choi, Seong-Hae Jeong, Seung-Han Lee, Jae-Myung Kim, Sang-Ho Kim, Hyo-Jung Kim, Kwang-Dong Choi, Ji-Soo Kim

**Affiliations:** 1 Department of Neurology, Pusan National University Hospital, Pusan National University School of Medicine and Biomedical Research Institute, Republic of Korea; 2 Department of Neurology, Jeonbuk National University Hospital & School of Medicine, Jeonju, Republic of Korea; 3 Department of Neurology, Keimyung University School of Medicine, Daegu, Republic of Korea; 4 Department of Neurology, Ulsan University Hospital, University of Ulsan College of Medicine, Ulsan, Republic of Korea; 5 Department of Neurology, Pusan National University School of Medicine, Research Institute for Convergence of Biomedical Science and Technology, Pusan National University Yangsan Hospital, Yangsan, Republic of Korea; 6 Department of Neurology, College of Medicine, Seoul National University, Seoul, Republic of Korea; 7 Department of Neurology, Clinical Neuroscience Center, Seoul National University Bundang Hospital, Seongnam, Republic of Korea; 8 Department of Neurology, Chungnam National University School of Medicine, Daejeon, Republic of Korea; 9 Department of Neurology, Chonnam National University Medical School, Gwangju, Republic of Korea; 10 Department of Neurology, College of Medicine, Dong-A University, Busan, Republic of Korea; 11 Biomedical Research Institute, Bundang Hospital, Seongnam, Republic of Korea; Universiti Malaya Fakulti Perubatan: University of Malaya Faculty of Medicine, MALAYSIA

## Abstract

**Background:**

Although new preventive treatments for migraine have emerged, it remains essential to validate the efficacy of established drugs to ensure broader therapeutic options for migraine and, in particular, for vestibular migraine where clinical evidence is more limited. This study aimed to assess the therapeutic effectiveness of nimodipine, an L-type calcium channel blocker, in patients with migraine and vestibular migraine, with reference to outcomes observed with topiramate.

**Methods:**

Using a prospective open-label study involving nine referral-based university hospitals in South Korea, we recruited 850 patients (81% women, mean age ± SD = 41 ± 12) with migraine, including 255 with vestibular migraine. The primary outcome was the change in headache days over three months. The secondary outcomes included changes in pain rating scale, Migraine Disability Assessment Scale (MIDAS) and Headache Impact Test-6 (HIT-6). The outcomes of vestibular migraine included dizziness days and intensity, Dizziness Handicap Inventory, and UCLA-Dizziness Questionnaire.

**Results:**

Of the 850 patients, 465 (55%) completed three months of evaluation (205 in the nimodipine group, 160 in the topiramate group, and 100 in the combination group). All groups showed a significant reduction in the headache days (1.2–2 days/week, *p* < 0.001) without inter-group differences (*p* = 0.865). The topiramate group showed greater improvements in MIDAS and HIT-6 scores than the nimodipine (*p* = 0.004) and combination groups (*p* = 0.040). For vestibular migraine (n = 131), all groups improved in headache and dizziness outcomes (*p* < 0.001) without inter-group differences. Adverse events leading to study discontinuation were observed only in 14 (2%) patients without a difference among the groups.

**Conclusion:**

Nimodipine was associated with improvements in headache-related outcomes in migraine and in both headache- and dizziness-related outcomes in vestibular migraine. **Given the observed improvements and favorable tolerability,** nimodipine may be a valuable treatment option for migraine and vestibular migrain.

**Trial registration**

cris.nih.go.kr (KCT0010555).

## Introduction

The introduction of anti-calcitonin gene-related peptide (CGRP) antibodies, a novel migraine-specific medication supported by robust clinical evidence, has marked a new era in migraine treatment [1]. Despite their groundbreaking potential, however, some patients show limited responses to anti-CGRP therapies, and their relatively high costs pose challenges to a widespread adoption, raising concerns regarding cost-effectiveness and accessibility [2,3]. Consequently, these limitations have spurred a renewed interest in reviving traditional migraine treatments to better define their role in contemporary migraine therapy [4,5].

Within this context, the calcium channel blockers (CCBs) have re-emerged as an option for prevention of migraine [2,4–6]. Nimodipine, a dihydropyridine calcium channel antagonist introduced in the 1980s, was initially recognized for its potential in the management of migraine. However, its adoption waned due to limited evidence from small-scale studies and development of newer therapeutic alternatives [7]. More recently, emerging evidence has highlighted its benefits in managing headache and dizziness, further underscoring its therapeutic utility [6,8,9]. These findings point to its potential relevance, [7] particularly in vestibular migraine—a condition where its efficacy remains underexplored. [9] In contrast, Topiramate is a well-established oral preventive therapy for migraine, supported by randomized trials and guideline recommendations, and has also been used in vestibular migraine with 25–100 mg/day in clinical practice [10–12]. Although a definitive minimum treatment duration for topiramate has not been established, studies in migraine populations have reported clinically meaningful reductions in headache frequency or intensity within approximately four weeks of treatment initiation [13,14].

The primary objective of this study was to evaluate the effectiveness of nimodipine in patients with migraine, including those with vestibular migraine, over a three-month follow-up. A secondary, exploratory objective was to compare outcomes observed with nimodipine, topiramate, and their combination in routine practice.

## Methods

### Design of the study

This prospective, open-label, multicenter observational study was conducted across nine referral centers in South Korea, targeting migraine patients who presented with headache or dizziness as the primary symptom from January 2020 to May 2024. The patients were assigned to either the nimodipine, topiramate, or combination group at the discretion of the treating physician. The combination group was included to reflect routine prescribing patterns. Analyses were conducted using a per-protocol approach, including only patients who completed the 3-month follow-up assessments and continued their medications throughout the study period. Patients who discontinued the study before the 3-month visit were excluded from the analyses.

This study followed the tenets of the Declaration of Helsinki and was performed according to the guidelines of Institutional Review Board of Pusan National University Hospital (1911-027-085). All patients were fully informed about the study, and written informed consent was obtained from each participant. The trial was registered at cris.nih.go.kr (KCT0010555) and the first registration date was 06/01/20 (https://cris.nih.go.kr/cris/search/detailSearch.do?seq=30149&search_page=L). The study protocol and CONSORT checklist are included as Supporting Information (S1 and S2 Files).

### Participants

Patients aged 18–75 years with a diagnosis of migraine, with or without aura, based on the International Classification of Headache Disorders, 3rd edition (ICHD-3), were eligible for inclusion if they had not used preventive migraine medications in the preceding year. Subgroup analysis was also performed for participants meeting the ICHD-3 diagnostic criteria for vestibular migraine. The patients diagnosed as vestibular migraine were evaluated and enrolled by board-certified neurologists with subspecialty training in neuro-otology. To ensure diagnostic accuracy for vestibular migraine, alternative neuro-otologic conditions including Ménière disease, vestibular neuritis, or benign paroxysmal positional vertigo were systematically excluded based on detailed neurological examination, bedside vestibular examination, and/or vestibular function tests when they indicated. Exclusion criteria included; 1) headache or dizziness attributable to another condition, such as central nervous system disorders; 2) presence of significant comorbidities, including liver or kidney diseases, malignancy, or other medical conditions that can hinder participation; 3) requirement for daily analgesic use for severe pain; 4) contraindications to CCBs or topiramate, including pregnancy; 5) use of other preventive migraine medications during the study period; 6) any other condition deemed by the investigators to impede participation or lack of patient consent.

Intermittent uses of analgesics (NSAIDs or acetaminophen) were permitted for acute severe headache. In patients with vestibular migraine, dimenhydrinate and metoclopramide were used only in cases of severe dizziness accompanied by vomiting. Other vestibular suppressants, including betahistine, meclizine, and benzodiazepines, were not used during the study period. Analgesics and dimenhydrinate were not used prophylactically and were administered only intermittently when symptoms were severe.

### Intervention

Nimodipine was administered 60 mg per day while topiramate was titrated from 25 mg to 100 mg per day according to individual responses. At enrollment, patients received a printed headache diary to document daily medication use, and headache occurrence and intensity. Patients with vestibular migraine also recorded dizziness frequency and intensity. Diaries were reviewed at each monthly visit when patients completed questionnaires and received prescriptions for the next month. The study terminated 3 months later. Patients were classified as dropouts if they withdrew consent, discontinued medication due to side effects, were considered noncompliant by the investigator, or missed more than one-third of prescribed medications over the month.

### Outcomes

The primary endpoint was the change in headache days per week from baseline to 3 months after treatment initiation. The secondary outcomes included headache intensity measured on a 0–10 Wong-Baker pain rating scale (PRS), migraine-related clinical burden assessed using the Headache Impact Test-6 (HIT-6), and disability evaluated with the Migraine Disability Assessment (MIDAS). The PRS and HIT-6 were assessed at the baseline, two and three months later while MIDAS was evaluated at the baseline and three months later. For patients with vestibular migraine, additional outcomes included changes in dizziness days per week and dizziness intensity (0–10 visual analogue scale, VAS). The Dizziness Handicap Inventory (DHI) and UCLA Dizziness Questionnaire (UCLA-DQ) were also assessed at same time.

### Statistical analyses

The sample size calculation was primarily conducted for the comparison between the two monotherapy groups. Assuming a power of 80% and a two-sided significance level of 0.05, a mean difference of 0.5 headache days per week, and an estimated standard deviation of 2 days, a minimum of 140 patients per group was required. The assumed effect size was chosen to clinically meaningful difference as the previous studies corresponding to approximately two headache-free days per month [14]. These assumptions were based on pragmatic considerations given the limited availability of prior comparative data in vestibular migraine. Because treatment allocation was investigator driven and non-randomized, with anticipated imbalance in group sizes, the study aimed to enroll a larger overall cohort to allow stable estimation of treatment effects across clinically relevant subgroups. The combination therapy group was included for exploratory purposes and was not incorporated into *priori* power calculation. Accordingly, the final enrollment target was determined pragmatically, taking into account expected dropout rates of 40–50% observed in routine clinical practice.

Statistical inference was prespecified for the primary endpoint only. Secondary and exploratory outcomes were interpreted descriptively. Where post-hoc pairwise comparisons were performed, p-values were adjusted using the Bonferroni method for reference.

For continuous variables, mean, SD, and confidence intervals (CI) were presented, and statistical analyses were performed using the t-test and Wilcoxon rank-sum test. For categorical variables, the chi-squared test or Fisher’s exact test was used. To determine interaction effects in repeated measures data, linear mixed-effects models (LMMs) were applied using restricted maximum likelihood estimation. The models included age, visit, group, and a group-by-visit interaction as fixed effects, and a subject-specific random intercept to account for repeated measures. Post-hoc comparisons were conducted to evaluate between-group differences at each visit and within-group changes over time. Bonferroni-adjusted significance thresholds were applied for inferential interpretation. Missing data were imputed using the Last Observation Carried Forward (LOCF) method to provide conservative estimates in this non-randomized setting.

Statistical analyses were performed using R version 4.3.3 (R Core Team, 2024; https://cran.r-project.org), with linear mixed-effects models fitted using the lme4 and lmerTest packages. A two-sided p-value < 0.05 was considered statistically significant.

## Results

### Demographics and clinical features

**Fig 1:**
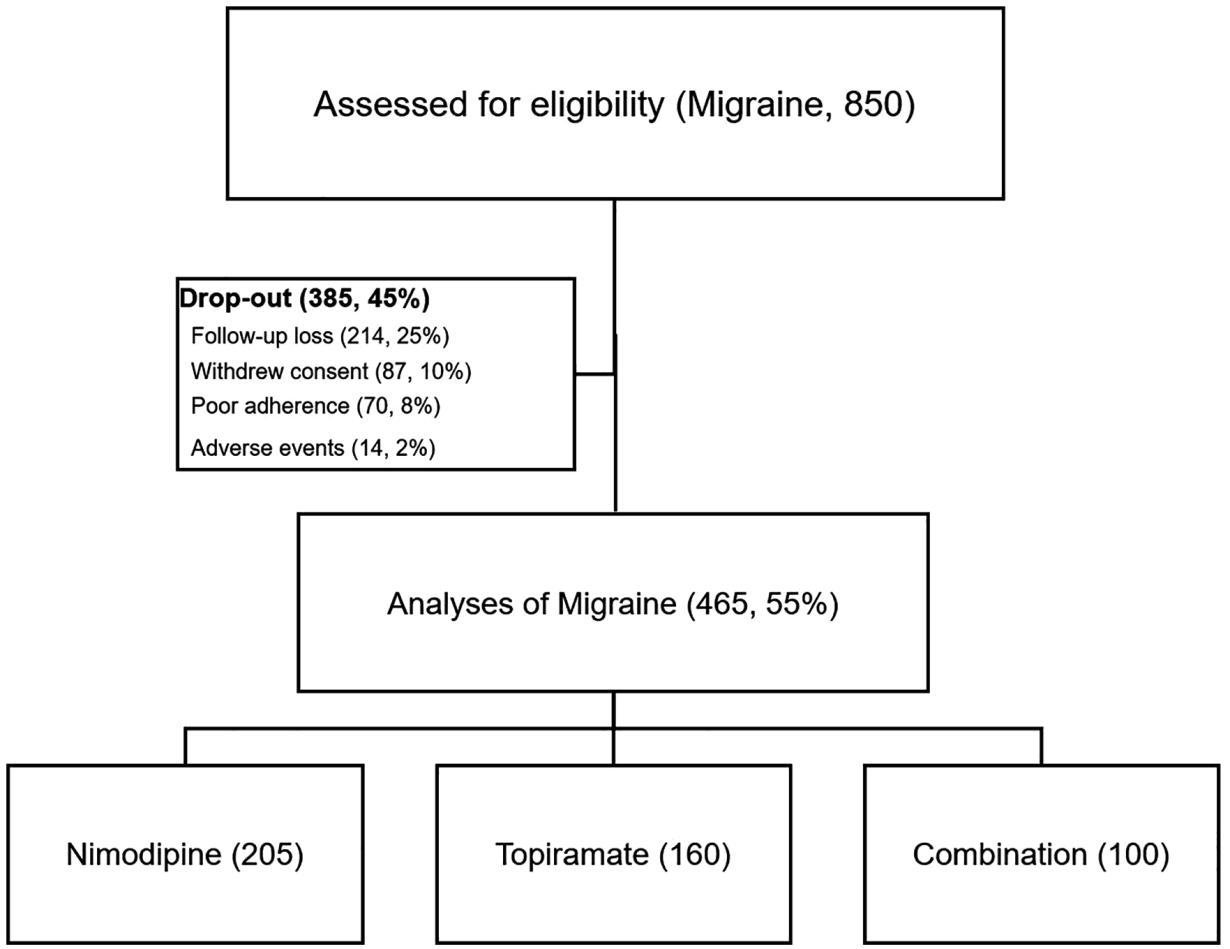
Study flow diagram of the migraine cohort.

A total of 850 patients (687 (81%) women, mean age ± SD = 41 ± 12) had been recruited with the first visit in February 2020 and the last in May 2024 (Fig 1). Of these, 465 patients (55%) completed the study. Among the 385 patients (45%) lost to follow-up, 301 (35%) withdrew their consent voluntarily, 70 (8%) were dropped out due to poor medication adherence (missing more than one-third of the prescribed medications during the preceding month), and 14 (2%) discontinued the study due to adverse events.

Adverse events leading to study discontinuation were reported in 14 patients (14/850, 2%), and included paresthesia in 9 (64%), gastrointestinal disturbances in 3 (21%), rash in one (7%), and other side effect in the remaining one (7%). The nimodipine group showed a lower tendency of side effects (2 in the nimodipine group, 8 in the topiramate group, 4 in the combination group; *p* = 0.056).

No significant differences were observed between the patients included and excluded regarding the age, sex, and proportion of vestibular migraine (S1 Table). In addition, the participants were evenly allocated to the three treatment groups (*p* = 0.085). The baseline scores of PRS, MIDAS, and HIT-6 did not differ between the patients included and excluded, but the frequency of headache was higher in the patients excluded (*p* = 0.013) (S1 Table).

### Analyses for migraine (total cohort)

Of the 465 patients who completed the follow-ups, 205 (44%) belonged to the nimodipine group, 160 (34%) to the topiramate group, and 100 (24%) to the combination group. Patients in the combination group were older (*p* < 0.001) and had more headache days (*p* = 0.008, Table 1). The scores of PRS, MIDAS, and HIT-6 were higher in the topiramate group (Table 1).

**Table 1:** Clinical characteristics and baseline scores in patients with migraine and vestibular migraine.

Migraine (n = 465)	Nimodipine (n = 205)	Topiramate (n = 160)	Combination (n = 100)	*p*-value
Age	44.1 (13.3)	37.2 (12.0)	45.1 (12.0)	<0.001
Sex, men (%)	40 (20)	26 (16)	17 (17)	0.699
Headache days per week, 0–7	3.6 (2.1)	3.4 (2.0)	4.2 (2.2)	0.008
Pain rating scale, 0–10	5.3 (2.2)	6.2 (2.3)	5.8 (2.4)	0.002
Migraine Disability Assessment, 0–270	42.8 (55.7)	63.2 (68.7)	51.4 (55.4)	0.007
Headache Impact Test-6, 0–78	59.3 (8.1)	63.04 (7.0)	59.7 (8.8)	<0.001
Vestibular migraine (n = 131)	Nimodipine (n = 61)	Topiramate (n = 38)	Combination (n = 32)	
Age	43.2 (12.2)	34.8 (11.7)	40.5 (10.5)	0.003
Sex, men (%)	5 (8.2)	7 (18.4)	3 (9.4)	0.273
Headache days for week, 0–7	3.46 (2.1)	3.79 (2.1)	4.06 (2.3)	0.423
Pain rating scale, 0–10	5.0 (2.0)	6.0 (2.2)	5.6 (2.2)	0.046
Migraine Disability Assessment, 0–270	54.5 (77.0)	74.6 (72.0)	53.9 (61.3)	0.346
Headache Impact Test-6, 0–78	59.8 (6.7)	62.1 (6.6)	61.8 (6.6)	0.178
Dizziness days for week, 0–7	4.0 (2.5)	4.1 (2.2)	4.4 (2.2)	0.751
Visual analogue scale, 0–10	5.8 (2.2)	6.0 (1.7)	5.2 (2.5)	0.252
UCLA Dizziness Questionnaire, 0–25	16.9 (3.7)	16.0 (3.8)	16.9 (4.3)	0.457
Dizziness Handicap Inventory, 0–100	38.3 (17.4)	35.6 (19.9)	35.5 (21.4)	0.720

Note: Data are mean (SD) or n (%) unless otherwise stated.

The primary endpoint, the headache days decreased by approximately 2 days per week in all groups with a mean decrease at 1.9 in the nimodipine group, 1.8 in the topiramate group and 1.9 in the combination group (*p-*values < 0.001 for all groups, S1 Fig A, Table 2). Significant reductions of secondary outcomes were also observed in PRS (2.4–2.6 points), MIDAS (23–40 points), and HIT-6 (9–12 points) scores in all three groups (S1 Fig B–D and Table 2).

**Table 2:** Primary and secondary outcomes after three months.

	Mean (SD) at baseline			Mean (SD) at 3 month			Mean change (95% CI) from baseline			*p*-values for mean change after 3 months		
Migraine (n = 465)	Nimodipine	Topiramate	combination	Nimodipine	Topiramate	Combination	Nimodipine	Topiramate	Combination	Nimodipine	Topiramate	Combination
Headache days for week, 0–7	3.58 (2.13)	3.41 (2.00)	4.22 (2.18)	1.68 (1.87)	1.63 (1.67)	2.28 (2.05)	−1.9 (−2.2 to −1.5)	−1.8 (−2.1 to −1.5)	−1.9 (−2.4 to −1.5)	<0.001	<0.001	<0.001
Pain rating scale, 0–10	5.3 (2.2)	6.2 (2.3)	5.8 (2.4)	2.95 (2.28)	3.59 (2.71)	3.18 (2.33)	−2.4 (−2.8 to −2.0)	−2.6 (−3.1 to −2.1)	−2.6 (−3.1 to −2.06)	<0.001	<0.001	<0.001
Migraine Disability Assessment, 0–270	42.8 (55.7)	63.2 (68.7)	51.4 (55.4)	19.48 (35.96)	22.37 (33.39)	25.09 (37.58)	−23.3 (−30.3 to −16.41)	−39.8 (−49.21 to −30.38)	−26.3 (−37.20 to −15.34)	<0.001	<0.001	<0.001
Headache Impact Test-6, 0–78	59.29 (8.09)	63.04 (6.95)	59.67 (8.78)	50.08 (9.80)	50.94 (11.06)	50.45 (10.18)	−9.2 (−10.65 to −7.77)	−12.1 (−13.90 to −10.31)	−9.2 (−11.15 to −7.29)	<0.001	<0.001	<0.001
Vestibular migraine (n = 131)	Nimodipine	Topiramate	combination	Nimodipine	Topiramate	Combination	Nimodipine	Topiramate	Combination	Nimodipine	Topiramate	Combination
Headache days for week, 0–7	3.46 (2.10)	3.79 (2.11)	4.06 (2.30)	2.10 (2.17)	1.80 (1.94)	2.82 (2.39)	−1.4 (−2.03 to −0.70)	−2.0 (−2.68 to −1.30)	−1.2 (−2.04 to −0.45)	<0.001	<0.001	0.002
Pain rating scale, 0–10	4.97 (2.01)	6.03 (2.16)	5.59 (2.15)	3.64 (2.35)	3.32 (2.45)	3.19 (2.24)	−1.3 (−1.98 to −0.68)	−2.7 (−3.80 to −1.62)	−2.4 (−3.34 to −1.47)	<0.001	<0.001	<0.001
Migraine Disability Assessment, 0–270	54.52 (76.93)	74.58 (71.99)	53.88 (61.27)	20.50 (37.99)	28.61 (41.61)	34.84 (50.40)	−34.02 (−51.34 to −16.71)	−45.97 (−64.31 to −27.64)	−19.03 (−44.19 to 6.13)	<0.001	<0.001	0.128
Headache Impact Test-6, 0–78	59.75 (6.74)	62.05 (6.57)	61.78 (6.63)	52.26 (9.62)	49.66 (10.92)	53.91 (8.58)	−7.49 (−9.66 to −5.33)	−12.39 (−15.93 to −8.86)	−7.88 (−10.60 to −5.15)	<0.001	<0.001	<0.001
Dizziness days for week, 0–7	4.04 (2.47)	4.07 (2.17)	4.41 (2.15)	2.09 (2.35)	1.78 (2.30)	2.49 (2.43)	−1.95 (−2.55 to −1.35)	−2.29 (−3.11 to −1.47)	−1.91 (−2.57 to −1.26)	<0.001	<0.001	<0.001
Visual analogue scale, 0–10	5.77 (2.22)	6.04 (1.72)	5.19 (2.51)	3.34 (2.62)	2.42 (2.47)	3.02 (2.45)	−2.43 (−3.31 to −1.54)	−3.62 (−4.55 to −2.69)	−2.17 (−3.22 to −1.12)	<0.001	<0.001	<0.001
UCLA Dizziness Questionnaire, 0–25	16.89 (3.66)	15.95 (3.79)	16.88 (4.32)	12.67 (4.93)	10.68 (5.47)	11.44 (4.44)	−4.21 (−5.30 to −3.12)	−5.26 (−6.88 to −3.65)	−5.44 (−7.17 to −3.71)	<0.001	<0.001	<0.001
Dizziness Handicap Inventory, 0–100	38.30 (17.39)	35.63 (19.93)	35.50 (21.41)	21.87 (18.41)	18.26 (20.09)	20.56 (19.49)	−16.43 (−21.25 to −11.61)	−17.37 (−23.97 to −10.77)	−14.94 (−21.96 to −7.92)	<0.001	<0.001	<0.001

**Fig 2:**
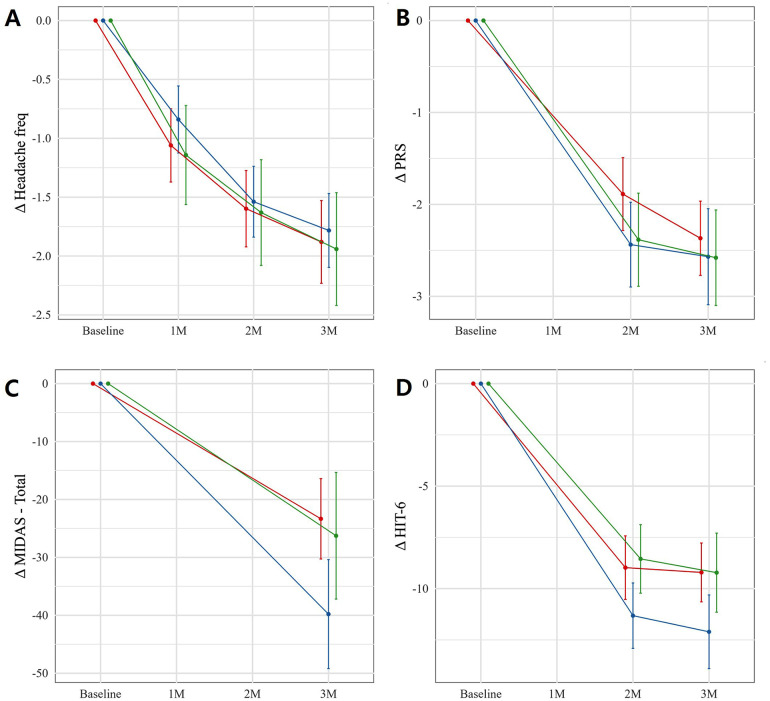
Changes (Δ) of the scores among the nimodipine, topiramate, and combination groups analyzed using a linear mixed model in the patients with migraine. The headache days (Headache freq, A) and Wong-Baker pain rating scale (PRS, B) decreased over three months in all groups (*p* < 0.001 for both) without differences among the groups (*p* = 0.945 and 0.368, respectively). The Migraine Disability Assessment (MIDAS, C) and Headache Impact Test-6 (HIT-6, D) scores showed reductions over three months in all groups (*p* < 0.001 for both), but significant differences were observed between-group differences (*p* = 0.004 and 0.040, respectively, S2 Table). (Dots (Dots represent model-estimated means; bars indicate 95% confidence intervals. Red = nimodipine, Blue = topiramate, Green = combination group).

Primary inter-group analyses focused on comparisons between the two monotherapy groups. No significant differences were observed between nimodipine and topiramate groups in the mean reduction of headache days (mean difference: −0.1, *p* = 0.691) or PRS scores (mean difference: 0.2, *p* = 0.543) (Table 3). Similary, no significant group differences were detected in these outcomes when all groups were compared (*p* = 0.945 and 0.368, respectively, Fig 2A and 1B). However, the decrease in MIDAS and HIT-6 scores differed among three groups (*p* = 0.004 and 0.040, respectively, Fig 2C and 1D, S2 Table). Post-hoc analyses (Table 3) showed greater reductions in MIDAS (16 points) and HIT-6 (3 points) scores in the topiramate group compared to the nimodipine group (*p* = 0.005 and 0.012, Table 3). The topiramate group also showed a greater HIT-6 reduction than the combination group (3 points, *p* = 0.038, Table 3). Temporal changes from baseline to the final visit were significant, while age did not significantly influence these changes (S2 Table).

**Table 3:** Mean differences between the groups between-group pairwise comparisons.

Migraine	Mean difference between nimodipine and topiramate (95% CI)	*p-*value	Mean difference between nimodipine and combination group (95% CI)	*p-*value	Mean difference between topiramate and combination group (95% CI)	*p-*value
Headache days for week, 0–7	−0.1 (−0.6 - 0.4)	0.691	0.1 (−0.5 - 0.7)	0.843	0.2 (−0.4 - 0.7)	0.569
Pain rating scale, 0–10	0.2 (−0.4 0.8)	0.543	0.2 (−0.5 - 0.9)	0.540	0 (−0.8 - 0.8)	0.977
Migraine Disability Assessment, 0–270	15.8 (4.4 27.2)	0.005	12.9 (−9.5 - 15.4)	0.644	−12.9 (−27.6 - 1.8)	0.070
Headache Impact Test-6, 0–78	2.9 (0.6 - 5.2)	0.012	0 (−2.4 - 2.5)	0.993	−2.9 (−5.6 - −0.2)	0.038
Vestibular migraine	Mean difference between nimodipine and topiramate (95% CI)	*p-*value	Mean difference between nimodipine and combination group (95% CI)	*p-*value	Mean difference between topiramate and combination group (95% CI)	*p-*value
Headache days for week, 0–7	0.6 (−0.4 - 1.6)	0.208	−0.1 (−1.2 - 1.0)	0.827	−0.8 (−1.8 - 0.3)	0.150
Pain rating scale, 0–10	1.4 (0.2 - 2.6)	0.021	1.1 (0 - 2.2)	0.056	−0.3 (−1.7 - 1.1)	0.675
Migraine Disability Assessment, 0–270	11.9 (−14.0 - 37.9)	0.364	−15.0 (−44.6 - 14.6)	0.318	−26.9 (−56.9 - 3.0)	0.077
Headache Impact Test-6, 0–78	4.9 (1.1 - 8.8)	0.013	1.8 (−3.2 - 3.9)	0.830	−4.5 (−9.0 − 0)	0.050
Dizziness days for week, 0–7	0.3 (−0.7 - 1.3)	0.498	0 (−1.0 - 0.9)	0.939	−0.4 (−1.4 - 0.7)	0.482
Visual analogue scale, 0–10	1.2 (−0.1 - 2.5)	0.077	−0.3 (−1.7 - 1.2)	0.723	−1.4 (−2.8 - −0.1)	0.039
UCLA Dizziness Questionnaire, 0–25	1.1 (−0.8 - 2.9)	0.264	1.2 (−0.7 - 3.2)	0.211	0.2 (−2.2 - 2.5)	0.882
Dizziness Handicap Inventory, 0–100	0.9 (−7.0 - 8.9)	0.814	−1.5 (−9.8 - 6.8)	0.721	−2.4 (−11.9 - 7.1)	0.611

### Analyses for vestibular migraine

Of the 131 patients with vestibular migraine (131/456, 28%) who completed the follow-ups, 61 (47%) belonged to the nimodipine group, 38 (29%) to the topiramate group, and 32 (24%) to the combination group (Table 2). Baseline comparisons showed younger patients in the topiramate group (*p* = 0.003) and lower PRS scores in the nimodipine group (*p* = 0.046, Table 2).

**Fig 3:**
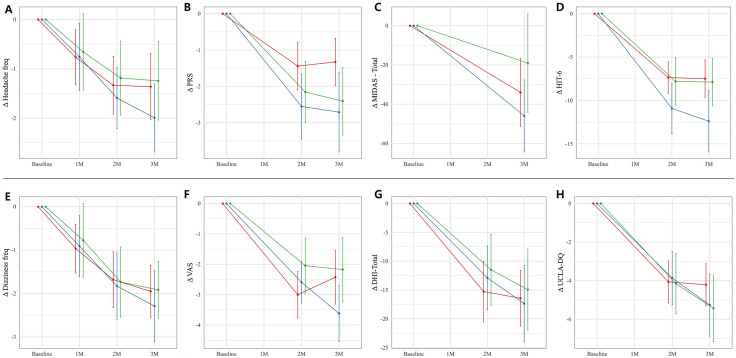
Changes (Δ) of the scores among the nimodipine, topiramate, and combination groups analyzed using a linear mixed model in the patients with vestibular migraine. For headache-related measures (A-D), significant reductions were observed for the headache days (A), PRS (B), MIDAS (C), and HIT-6 (D) scores (*p* < 0.001 for all). Between-group comparisons showed no significant differences for headache days, PRS, and MIDAS (*p* = 0.544, 0.052, and 0.179, respectively, S3 Table), while HIT-6 scores significantly differed between groups (*p* = 0.041, S3 Table). For dizziness-related measures **(E – H)**, including dizziness frequency **(E)**, visual analogue scale for dizziness intensity (VAS, **F)**, Dizziness Handicap Inventory (DHI, **G)**, and UCLA Dizziness Questionnaire (UCLA-DQ, H) scores, all groups demonstrated significant reductions over the 3-month period (*p* < 0.001 for all, S3 Table), with no significant differences among groups (S3 Table). (Dots represent model-estimated means; bars indicate 95% confidence intervals. Red = nimodipine, Blue = topiramate, Green = combination group).

Headache-related outcomes improved significantly in all three groups, with reductions in the headache days per week (1.2–2 days/week), and PRS (1–3 points) and HIT-6 scores (9–12 points) (S1 Fig E, F, and H, Table 2). The MIDAS scores also improved significantly in the nimodipine and topiramate groups (*p* < 0.001 for both), but not in the combination group (*p* = 0.128, S1 Fig G, Table 2). The dizziness-related scores, including the dizziness days per week, VAS, DHI, and UCLA-DQ, showed significant reductions in all three groups (*p* < 0.001, Table 2 and S1 Fig I–L). Consistent with the primary analytic strategy, inter-group comparisons focused on the two monotherapy groups. Inter-group comparisons showed no significant differences in reductions of headache days, PRS, or MIDAS scores among the groups (Fig 3A-3C). However, the topiramate group showed a greater HIT-6 (5 points, *p* = 0.013) and PRS (1 points, *p* = 0.021) reductions than the nimodipine (Table 3). For dizziness-related outcomes, no significant differences were observed among the groups (Fig 3E–3H) even though the post-hoc pairwise comparisons indicated a greater VAS reduction (1 points) in the topiramate group than in the combination group (*p* = 0.039, Table 3). Temporal changes from baseline to the final visit were significant, while age did not significantly influence these changes (S3 Table).

### Imputation analysis

An additional analysis using LOCF method was performed to address missing data from patients lost to follow-up (n = 385, 45%). Overall, results were largely consistent with the primary complete-case linear mixed model analysis for most outcomes based on percent changes from baseline

Among the 850 patients with migraine, percent changes in headache frequency, PRS, and HIT-6 scores decreased significantly across all treatment groups (all *p* < 0.001; S2 Fig A, B, and D). In contrast, percent changes in MIDAS scores did not show significant improvement following imputation, and no significant between-group differences were detected (*p* > 0.05; S2 Fig C).

Similarly, among the 255 patients with vestibular migraine, the percent changes in headache frequency, PRS, and HIT-6 scores significantly decreased in all groups (all *p* < 0.001; S3 Fig A, B, and D). In contrast, the percent change in MIDAS scores showed no improvement from the baseline (*p* = 0.931; S3 Fig C). No significant differences were observed among the treatment groups for any of the measured outcomes (S3 Fig 3).

## Discussion

The key findings of this study describe longitudinal changes in headache- and dizziness-related outcomes observed during nimodipine treatment in patients with migraine and vestibular migraine. Over the three-month follow-up, reductions in headache frequency and intensity were observed in the nimodipine group, along with improvements in dizziness-related outcomes among patients with vestibular migraine. The overall scores of symptom improvement observed in the nimodipine group was comparable to that observed in patients receiving topiramate. Nimodipine was also associated with fewer adverse events, suggesting a potentially more favorable tolerability profile although this difference did not reach statistical significance (*p* = 0.056).

Although standardized primary endpoints have not been firmly established for pharmacological studies in migraine, [15] headache days and intensity are commonly used as primary outcomes in clinical trials. [14,15] In this context, the 50% reduction in headache frequency from four to two days in the nimodipine group represents a relative improvement. [14] Patient-reported outcome measures such as MIDAS and HIT-6, which are widely used as primary or secondary endpoints in migraine studies, [15] also showed notable improvement in both the nimodipine and topiramate groups. Previous studies have suggested a minimally important between-group difference of 2.3 points for HIT-6, [16] so the observed difference of 2.9 points between treatment groups therefore suggests a relevant change. Although a formal minimally important difference has not been established for MIDAS, prior studies commonly use a score of 40 to define severe disability and the observed shift from scores above this threshold to lower values in both groups can indicates a meaningful reduction in disease burden. Thresholds for patient-reported dizziness outcomes in vestibular migraine remain less clearly defined. Nevertheless, a Dizziness Handicap Inventory score of approximately 30 has been proposed to distinguish mild from moderate dizziness, [17] suggesting that the degree of improvement observed in our results is likely to be perceptible to patients.

Topiramate is a well-established preventive medication for migraine. [18,19] although its use is contraindicated during pregnancy and requires caution in women of childbearing age. [18] Despite these limitations, reports have demonstrated its efficacy in managing dizziness associated with migraine, [20] suggesting its potential utility in treating vestibular migraine as well. [12] Randomized controlled trials have also shown significant improvement in headache outcomes within one month of treatment initiation in migraine populations, [13] supporting the use of a three-month primary endpoint in the present study, despite differences in dose escalation and titration schedules compared with nimodipine. In our cohort, topiramate was associated with improvements in both headache- and dizziness-related outcomes. These observations support its continued use as a treatment option for vestibular migraine in routine practice [12,13].

CCBs are one of the classic migraine medications currently undergoing reevaluation. There is a renewed interest in these traditional medications [5,19]. A recent review of 50 clinical trials on the efficacy of blood pressure-lowering drugs for prevention of migraine demonstrated that several classes of antihypertensive medications including alpha-blockers, beta-blockers, ACE inhibitors, and CCBs, could effectively reduce the headache days by approximately 0.7 to 1.8 per month. [21] Among these, CCBs showed the greatest reduction, with an average decrease of 1.8 days per month, highlighting their potential utility in migraine prophylaxis [4].

Nimodipine, a dihydropyridine calcium channel blocker (CCB), primarily targets L-type voltage-gated calcium channels. While the exact mechanism of its efficacy on migraine remains unclear, nimodipine appears to modulate cortical spreading depolarization (CSD), thereby reducing neurovascular coupling disruptions and metabolic stress. [7] Additionally, recent mouse studies suggest that nimodipine accelerates cerebral blood flow recovery during spreading oligemia, further supporting its potential as a preventive medication for migraine. [6] Despite nimodipine is often categorized as “not rated” [21] or “undetermined” [18] in the current treatment guidelines for migraine since only small-scale studies are available, our findings suggest that nimodipine may warrant consideration as an additional preventive option in migraine.

Previous clinical studies, though limited in scale, have demonstrated a meaningful efficacy of nimodipine in migraine prophylaxis. [22–24] A randomized, double-blind study found that nimodipine could reduce the headache frequency by 40% compared to 14% with placebo over three months. [22] Similarly, another trial with 60 participants showed a 70% improvement in headache symptoms with nimodipine (120 mg/day) versus 30% with placebo over 13 weeks, with no severe adverse effects. [23] A study of 35 patients with migraine and cluster headache found that nimodipine (60 mg/day and 120 mg/day) significantly reduced headache frequency, with greater efficacy for classic migraine than for cluster headache. [24] Higher doses nimodipine achieved a 71% reduction in headache frequency compared to 55% at lower doses (*p* < 0.01). Additionally, nimodipine had fewer adverse effects than nifedipine or verapamil and was not associated with severe adverse effects [24].

However, a relatively large-scale randomized double-blind study comparing nimodipine with placebo over three months in patients with migraine with and without aura found a similar efficacy between the two groups [25,26]. In the nimodipine group, the migraine index scores improved by approximately 60% for both migraine with (n = 33) and without aura (n = 76). Of interest, in the placebo group, the migraine index scores also improved by 60% in the migraine with aura group (n = 39) and by 75% in the migraine without aura group (n = 85) [25,26]. While the studies did not specify the composition of the placebo tablet, the results suggest that placebo alone may significantly contribute to prevention of migraine, warranting careful interpretation of these findings.

Since L-type calcium channels play a crucial role in signal transmission in vestibular and auditory hair cells, [9] selective L-type CCBs, such as nimodipine, flunarizine, and cinnarizine, have been widely used in managing not only migraine but also vestibular disorders. [8,9,12,27,28] Although it remains unclear whether these medications are more effective for headache or dizziness, they are expected to provide a benefit for the patients with vestibular migraine. [12] While no clinical trials have yet attempted nimodipine in VM, flunarizine 10 mg/day for 12 weeks significantly decreased the frequency and severity of vertiginous episodes in 52 patients with vestibular migraine even though headache did not respond. [29] In a randomized trial comparing flunarizine (10 mg daily), venlafaxine (37.5 mg daily), and sodium valproate (500 mg twice daily), flunarizine did not show superior efficacy over the other medications. [30] Nonetheless, a before-and-after comparison within the flunarizine group revealed significant reductions in the severity and frequency of vertigo, and DHI scores. [30] Our study demonstrated that nimodipine effectively improved both headache and dizziness in patients with vestibular migraine, highlighting its potential as a dual-target treatment option for this condition.

Our study has several limitations. First, its non-randomized, open-label design is inherently susceptible to bias, particularly given the subjective nature of several outcome measures. Randomization and blinding are essential to minimize placebo effects and expectation bias in these conditions; however, implementation of a placebo-controlled design across multiple centers was not feasible because of practical constraints. Consequently, treatment allocation was determined by treating physicians, which may have introduced selection bias and should be carefully considered when interpreting the results. These limitations are inherent to multicenter observational studies. Treatment allocation was influenced by clinician judgment, and patients with greater symptom severity or specific clinical characteristics may have been preferentially assigned to certain treatment arms. As a result, confounding by indication represents a major threat to internal validity, and the findings should be interpreted as comparative observations from routine clinical practice rather than as evidence of causal treatment effects.

Second, results from the combination therapy group should be interpreted with caution. As specified in the methods, analyses involving this group were exploratory and were not based on a prespecified hypothesis or statistical framework. Although combination therapy was clinically assumed to provide additive benefit, the observed outcomes did not demonstrate superior efficacy compared with monotherapy. Nevertheless, given that no single medication currently holds a priority in the treatment of vestibular migraine, [12,31] and treatment selection should be tailored to the clinical characteristics of individual patients, [31] this study design reflects real-world practice and offers insights into practical treatment approaches.

The relatively high dropout rate represents an important limitation. Because only patients who completed the study were included in the per-protocol analysis, the results may be subject to attrition bias and may not fully represent the entire study population.

Finally, discrepancies observed in MIDAS scores following last observation carried forward (LOCF) imputation highlight methodological challenges related to missing data. Given the substantial inter- and intra-individual variability of MIDAS scores across visits, imputation may have reduced statistical sensitivity for detecting treatment-related changes. Accordingly, MIDAS results should be interpreted cautiously. Nevertheless, the consistency of other outcome measures between the imputed and original analyses supports the overall robustness of the study’s conclusions.

Over three months of follow-up, nimodipine was associated with improvements in headache-related outcomes in migraine and in both headache- and dizziness-related outcomes in vestibular migraine. Given the observed improvements and favorable tolerability, nimodipine may be a valuable treatment option for migraine and vestibular migraine.

## Supporting information

S1 File Study protocol. (PDF)

S2 File CONSORT 2010 checklist. (DOCX)

S1 Table Comparisons between the patients included for and excluded from the analyses. (DOCX)

S2 Table Type III tests of fixed effects from linear mixed-effects models for primary and secondary outcomes in patients with migraine. (DOCX)

S3 Table Type III tests of fixed effects from linear mixed-effects models for primary and secondary outcomes in patients with vestibular migraine. (DOCX)

S1 Fig Changes in the frequency and intensity of headache in patients with migraine and in the frequency and severity of dizziness in those with vestibular migraine. The headache days per week (A), and the scores of Wong-Baker pain rating scale (PRS, B), Migraine Disability Assessment (MIDAS-Total, C), and Headache Impact Test-6 (HIT-6, D) decreased significantly with medications in all treatment groups. Patients with vestibular migraine also showed improvements in, the headache frequency (E) and intensity (PRS, F), MIDAS-Total (G), HIT-6 (H), dizziness days per week (I), visual analogue scale for dizziness intensity (VAS, J), Dizziness Handicap Inventory (DHI, K), and UCLA Dizziness Questionnaire (UCLA-DQ, L) scores with 3 months of medication (Dots represent the mean, and bars indicate the 95% CI. Red = nimodipine, Blue = topiramate, Green = combination group).
(PNG)

S2 Fig The percentage changes in clinical outcome scores in patients with migraine (n = 850), analyzed using a linear mixed model with Last Observation Carried Forward imputation. Headache frequency (A) and Wong-Baker Pain Rating Scale (PRS, B) scores significantly decreased over the three-month period in all groups (both *p* < 0.001), with no significant differences between groups (*p* = 0.870 and 0.727, respectively). In contrast, the Migraine Disability Assessment (MIDAS-total, C) score did not show a significant percent change from baseline (*p* = 0.604), nor were there significant inter-group differences (*p* = 0.139). Headache Impact Test-6 (HIT-6, D) scores decreased significantly in all groups (p < 0.001), with a significant difference observed among the three groups (*p* = 0.042). (Dots represent the mean and bars indicate the 95% CI. Red = nimodipine, Blue = topiramate, Green = combination group).
(PNG)

S3 Fig The percentage change in clinical outcome scores in patients with vestibular migraine (n = 255), analyzed using a linear mixed model with Last Observation Carried Forward imputation. For headache-related measures (A–D), significant percent reductions from baseline were observed in headache frequency (A), Wong-Baker Pain Rating Scale (PRS, B), and Headache Impact Test-6 (HIT-6, D) scores (all *p* < 0.001). However, no significant percent change was observed for the MIDAS-total score (C; *p* = 0.931). No significant inter-group differences were found for any of these measures (% change in headache frequency: *p* = 0.499; PRS: *p* = 0.238; MIDAS-total: *p* = 0.053; HIT-6: *p* = 0.050). For dizziness-related measures (E–H) including percent change of dizziness frequency (E), visual analogue scale for dizziness intensity (VAS, F), Dizziness Handicap Inventory (DHI-Total, G), and UCLA Dizziness Questionnaire (UCLA-DQ, H), all groups showed significant reductions over three months (all *p* < 0.001), with no significant inter-group differences (% change in dizziness frequency: *p* = 0.543; VAS: *p* = 0.064; DHI-Total: *p* = 0.373; UCLA-DQ: *p* = 0.501). (Dots represent the mean and bars indicate the 95% CI. Red = nimodipine, Blue = topiramate, Green = combination group).
(PNG)
