# Supplementary material for: Therapeutic efficacy of nimodipine and topiramate on migraine and vestibular migraine; A prospective multicenter open-label study
Source: PLoS One. 2026 Mar 19;21(3):e0344948. doi: 10.1371/journal.pone.0344948 (PMC13001945; doi:10.1371/journal.pone.0344948)
Supplement: S1 File — (PDF) [file pone.0344948.s001.pdf]

Therapeutic efficacy of nimodipine and topiramate on migraine and vestibular migraine: Comparison using a prospective multicenter open-label study

January 8, 2020

Ver 1.1

Seoyoung Choi, Department of Neurology, Pusan National University

Institutions and researchers

- **[Principal Investigator].**
  - Kim Ji Soo Bundang Seoul National University Hospital
  - **Kwang Dong Choi_Pusan National University Hospital**
- **[co-investigator].**
  - Kim Hyun Ah Kye Myung University Dongsan Medical Center
  - Kim Sang Ho Dong-A University Hospital
  - Seo Young Choi, Busan National University Hospital_Research Related Affairs
  - Jung Yoon Choi Bundang Seoul National University Hospital
  - Eun Hye Oh Yangsan Busan National University Hospital
  - Jiyoon Park Ulsan National University Hospital
  - Seung Han Lee, Chonnam National University Hospital
  - Sun Young Oh Chonbuk National University Hospital
  - Sung Hae Jung Chungnam National University Hospital

Research overview

- Enroll subjects who meet diagnostic criteria **for migraine** after neurologic examination and vestibular function testing. Individuals who meet the diagnostic criteria for vestibular migraine will be categorized into subgroups.
- BASELINE VISIT: Complete the headache questionnaire. The vestibular migraine group will complete an additional dizziness questionnaire. Any abnormalities in vestibular function testing will be noted in the e-CRF.
- The drug will be used as 1. nimodipine only 2. topiramate only 3. topiramate +nimodipine. Write the dose.
- Subject comes in once a month and submits a headache diary (to check compliance and frequency)
- The 2-month survey is in case the dropout rate is higher than expected.
- Check survey results after 3 months. In the vestibular migraine group, FU if vestibular function tests were abnormal.

Outcome and variables

- Primary outcome: To determine the effectiveness of Nimodipine in migraine
  - Notice a decrease in the frequency of headaches
  - Variable: Number of headaches /week

- Secondary outcome
  - Determine the effect of nimodipine on headache intensity and QoL in migraine (PRS, MIDAS, HIT-6)

  - Confirming the effectiveness of nimodipine and topiramate in vestibular migraine
    - Determine the intensity and severity of dizziness and its impact on QoL (VAS, DHI, UCLA-DQ)
    - Compare vestibular function before and after medication if it is abnormal
      - VOG VEMPs SVV vHIT

Migraine and Vestibular migraine

Exclusion:

- headache or dizziness attributable to another condition, such as central nervous system disorders
- presence of significant comorbidities : liver or kidney diseases, malignancy, or other medical conditions
- requirement for daily analgesic use for severe pain
- contraindications to CCBs or topiramate
- pregnancy
- use of other preventive migraine medications during the study period
- any other condition deemed by the investigators to impede participation or lack of patient consent.

✓ **Headache Frequency (/w)**
✓ Headache scores: PRS, MIDAS, HIT-6

✓ SUBGROUP: Vestibular migraine
  - ✓ VOG/HIT/VEMP/SVV
  - ✓ Dizziness Frequency (/w)
  - ✓ Dizziness scores: VAS, DHI, UCLA-DQ

Baseline,
ENROLL day + 3d

- Medication
    1. Nimodipine 1T bid
    2. Topiramate 25mg to 50mg /day
    3. Nimodipine + topiramate
- Headache Diary

Go to the second page

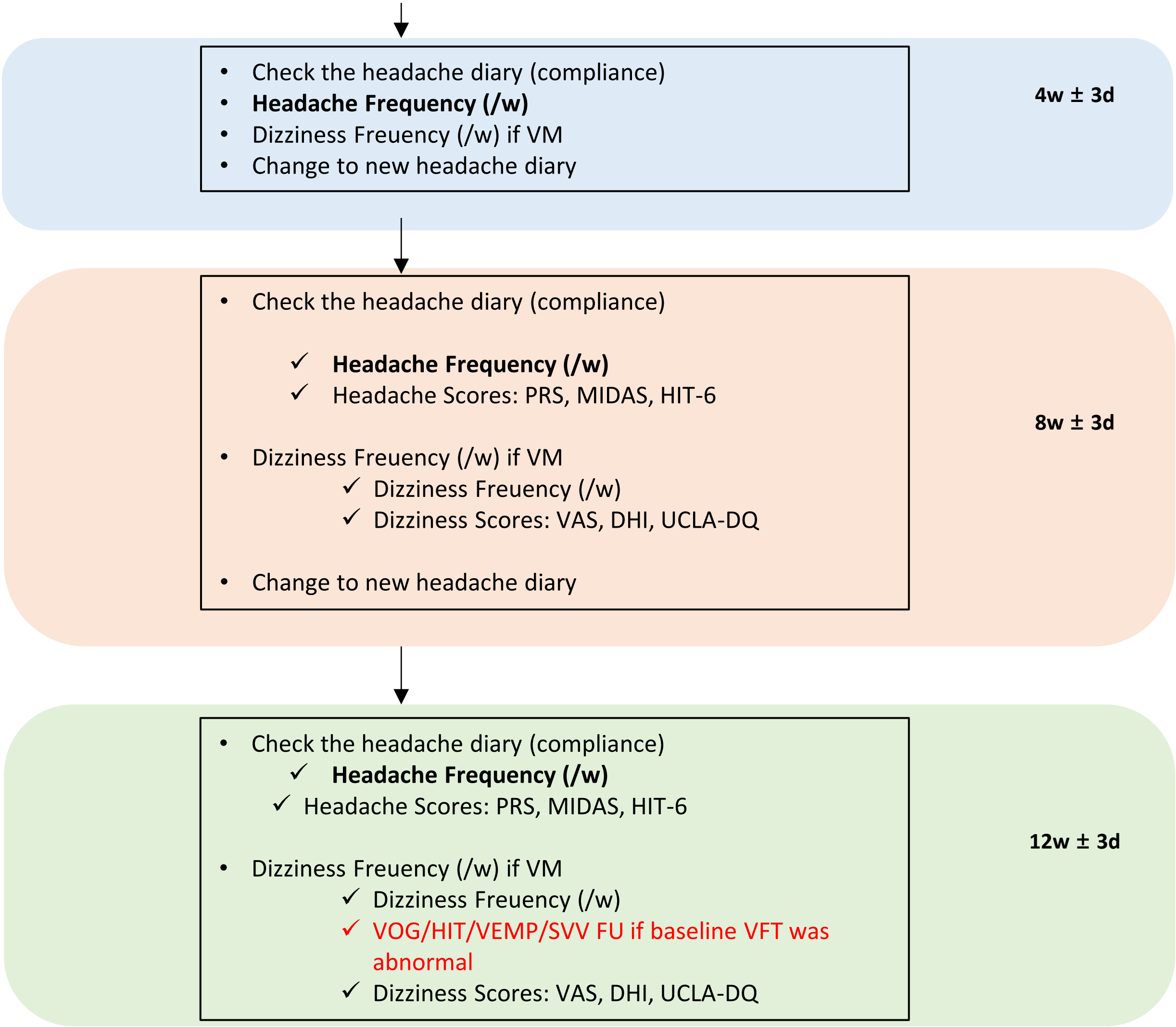

Check the headache diary (compliance)
Headache Frequency (/w)
Dizziness Freuency (/w) if VM
Change to new headache diary
4w ± 3d
Check the headache diary (compliance)
Headache Frequency (/w)
Headache Scores: PRS, MIDAS, HIT-6
Dizziness Freuency (/w) if VM
Dizziness Freuency (/w)
Dizziness Scores: VAS, DHI, UCLA-DQ
Change to new headache diary
8w ± 3d
Check the headache diary (compliance)
Headache Frequency (/w)
Headache Scores: PRS, MIDAS, HIT-6
Dizziness Freuency (/w) if VM
Dizziness Freuency (/w)
VOG/HIT/VEMP/SVV FU if baseline VFT was abnormal
Dizziness Scores: VAS, DHI, UCLA-DQ
12w ± 3d

Sample size

- IRB research protocol: 850 participants, described as an observational study based on previous research

- Stat. **(by HJ Kim, PhD.)**
  - Using G power
  - F tests - ANOVA: Repeated measures, between factors
    Analysis: A priori: Compute required sample size
  - Input
    - Effect size f = 0.1
      α err prob = 0.05
      Power (1-β err prob) = 0.80
      Number of groups = 3
      Number of measurements = 4
      Corr among rep measures = 0.5
  - Output: n = 606
  - 40% dropout rate
  - **Total n = 850**

Attention!

- SSRIs and TCAs are prohibited in general
- All PRN medications can be taken no more than one week per month
  - All digestive, painkillers, antispasmodics, anti-emetic, etc. including Bonaling/Alpram/Diazepam/NSAIDs/Macperan
- No beta blockers, drop out if you use them
- Botox users should not be enrolled.
- When the number of medication days (count) was 2/3 or less per month, drop-out
- drop out if you change the medication you were on at the time of registration

Note

- Researchers from the host institution may contact the center from time to time.
- Please provide the contact number and email of the person who will be directly responsible for the research at your center.
- The IRB protocol is different from the actual research methods. We will email you the IRB documents separately, so you can customize them for your institution and get IRB approval.

Subject No. Assignment (Institution Alphabetical)

1. Kyemyung University Dongshan Medical Center (starting from 1001)
2. Dong-A University Hospital (since 2001)
3. Pusan National University Hospital (starting at 3001)
4. Seoul National University Bundang Hospital (starting at 4001)
5. Yangsan Busan National University Hospital (starting from 5001)
6. Ulsan National University Hospital (starting at 6001)
7. Chonnam National University Hospital (starting at 7001)
8. Chonbuk National University Hospital (starting at 8001)
9. Chungnam National University Hospital (starting from 9001)

Stickers will be distributed, but please be aware that some may be lost.

Diagnostic criteria of migraine

1. At least five attacks[1] fulfilling criteria B-D
2. Headache attacks lasting 4-72 hr (untreated or unsuccessfully treated)[2;3]
3. Headache has at least two of the following four characteristics:
    1. unilateral location
    2. pulsating quality
    3. moderate or severe pain intensity
    4. aggravation by or causing avoidance of routine physical activity (eg, walking or climbing stairs)
4. During headache at least one of the following:
    1. nausea and/or vomiting
    2. photophobia and phonophobia
5. Not better accounted for by another ICHD-3 diagnosis.

A. At least two attacks fulfilling criteria B and C
B. One or more of the following fully reversible aura symptoms:

1. visual
2. sensory
3. speech and/or language
4. motor
5. brainstem
6. retinal

C. At least three of the following six characteristics:

1. at least one aura symptom spreads gradually over ≥5 minutes
2. two or more aura symptoms occur in succession
3. each individual aura symptom lasts 5-60 minutes[1]
4. at least one aura symptom is unilateral[2]
5. at least one aura symptom is positive[3]
6. the aura is accompanied, or followed within 60 minutes, by headache

- D. Not better accounted for by another ICHD-3 diagnosis.

Diagnostic criteria of vestibular migraine

A. At least **five episodes** fulfilling criteria C and D
B. Current or past history of **migraine with or without aura**
C. Vestibular symptoms of **moderate or severe intensity**, lasting between **5 minutes and 72 hours**
D. At least **50% of episodes** are associated with at least **one** of the following **migraine features**:

1. Headache with at least **two** of the following characteristics:
   1. Unilateral location
   2. Pulsating quality
   3. Moderate or severe intensity
   4. Aggravation by routine physical activity
2. Photophobia and phonophobia
3. Visual aura

E. Not better accounted for by another vestibular or ICHD-3 diagnosis
