# Supplementary material for: Therapeutic efficacy of nimodipine and topiramate on migraine and vestibular migraine; A prospective multicenter open-label study
Source: PLoS One. 2026 Mar 19;21(3):e0344948. doi: 10.1371/journal.pone.0344948 (PMC13001945; doi:10.1371/journal.pone.0344948)
Supplement: S1 Table — (DOCX) [file pone.0344948.s003.docx]

**Supplementary Table**

S1 Table. Comparisons between the patients included for and excluded from the analyses

| **Variable** | | **Included (n = 465)** | **Excluded (n = 385)** | ***p-*value** |
| --- | --- | --- | --- | --- |
| Age (years) | | 42.0 (13.0) | 40.3 (12.5) | 0.064 |
| Gender | Women | 382 (82) | 305 (79) | 0.321 |
| Drug | Nimodipine | 205 (44) | 158 (41) | 0.085 |
|  | Topiramate | 160 (34) | 119 (31) |  |
|  | Combination | 100 (22) | 108 (28) |  |
| Disease | Migraine | 334 (72) | 261 (68) | 0.229 |
|  | Vestibular migraine | 131 (28) | 124 (32) |  |
| Headache days per week, 0 - 7 | | 3.7 (2.1) | 4.1 (3.2) | 0.013 |
| Pain rating scale, 0 - 10 | | 5.7 (2.3) | 6.0 (2.3) | 0.070 |
| Migraine Disability Assessment, 0 - 270 | | 51.7 (61.0) | 51.5 (64.1) | 0.975 |
| Headache Impact Test-6, 0 - 78 | | 60.7 (8.1) | 61.5 (8.4) | 0.124 |

Note: Data are mean (SD) or n (%) unless otherwise stated.
