# Supplementary material for: Therapeutic efficacy of nimodipine and topiramate on migraine and vestibular migraine; A prospective multicenter open-label study
Source: PLoS One. 2026 Mar 19;21(3):e0344948. doi: 10.1371/journal.pone.0344948 (PMC13001945; doi:10.1371/journal.pone.0344948)
Supplement: S2 Table — (DOCX) [file pone.0344948.s004.docx]

**Supplementary Table**

**S2 Table. Type III tests of fixed effects from linear mixed-effects models for primary and secondary outcomes in patients with migraine.**

|  |  | Sum Sq | Mean Sq | NumDF | DenDF | F value | *p-*value |
| --- | --- | --- | --- | --- | --- | --- | --- |
| Change of Headache Frequency | age | 5.16 | 5.158 | 1 | 460.86 | 2.7454 | 0.098 |
|  | visit | 882.68 | 220.67 | 4 | 920.96 | 117.451 | <0.001 |
|  | drug | 0.21 | 0.107 | 2 | 460.68 | 0.0571 | 0.945 |
|  | visit:drug | 3.72 | 0.62 | 6 | 1383.2 | 0.33 | 0.921 |
| Change of Wong-Baker pain rating scale | age | 0.07 | 0.07 | 1 | 461 | 0.0172 | 0.896 |
|  | visit | 1685.25 | 561.75 | 3 | 692 | 146.5771 | <0.001 |
|  | drug | 8.33 | 4.16 | 2 | 461 | 1.0866 | 0.338 |
|  | visit:drug | 16.47 | 4.12 | 4 | 924 | 1.0741 | 0.368 |
| Change of the Migraine Disability Assessment scores | age | 5066 | 5066 | 1 | 922 | 3.3779 | 0.066 |
|  | visit | 189734 | 94867 | 2 | 922 | 63.2549 | <0.001 |
|  | drug | 16360 | 8180 | 2 | 922 | 5.4543 | 0.004 |
|  | visit:drug | 12859 | 6430 | 2 | 922 | 4.2871 | 0.014 |
| Change of Headache Impact Test-6 scores | age | 46.1 | 46.1 | 1 | 461 | 0.9159 | 0.339 |
|  | visit | 29863.4 | 9954.5 | 3 | 692 | 197.8977 | <0.001 |
|  | drug | 326.1 | 163.1 | 2 | 461 | 3.2415 | 0.040 |
|  | visit:drug | 522.6 | 130.7 | 4 | 924 | 2.5976 | 0.035 |
