# Supplementary material for: Therapeutic efficacy of nimodipine and topiramate on migraine and vestibular migraine; A prospective multicenter open-label study
Source: PLoS One. 2026 Mar 19;21(3):e0344948. doi: 10.1371/journal.pone.0344948 (PMC13001945; doi:10.1371/journal.pone.0344948)
Supplement: S3 Table — (DOCX) [file pone.0344948.s005.docx]

**Supplementary Table**

**S3 Table. Type III tests of fixed effects from linear mixed-effects models for primary and secondary outcomes in patients with vestibular migraine.**

|  |  | Sum Sq | Mean Sq | NumDF | DenDF | F value | *p-*value |
| --- | --- | --- | --- | --- | --- | --- | --- |
| Change of Headache Frequency | age | 1.121 | 1.121 | 1 | 126.57 | 0.5562 | 0.457 |
|  | visit | 178.354 | 44.588 | 4 | 253.5 | 22.1186 | <0.001 |
|  | drug | 2.464 | 1.232 | 2 | 126.64 | 0.6111 | 0.544 |
|  | visit:drug | 8.225 | 1.371 | 6 | 382.79 | 0.68 | 0.666 |
| Change of Wong-Baker pain rating scale | age | 0.14 | 0.141 | 1 | 127 | 0.0432 | 0.836 |
|  | visit | 368.79 | 122.931 | 3 | 190.99 | 37.8113 | <0.001 |
|  | drug | 19.68 | 9.839 | 2 | 127 | 3.0263 | 0.052 |
|  | visit:drug | 28.75 | 7.187 | 4 | 256 | 2.2105 | 0.068 |
| Change of the Migraine Disability Assessment scores | age | 1777 | 1777 | 1 | 127.4 | 0.842 | 0.361 |
|  | visit | 67148 | 33574 | 2 | 127.9 | 15.9122 | <0.001 |
|  | drug | 7350 | 3675 | 2 | 127.4 | 1.7417 | 0.179 |
|  | visit:drug | 6307 | 3154 | 2 | 128.41 | 1.4946 | 0.228 |
| Change of Headache Impact Test-6 scores | age | 14.3 | 14.25 | 1 | 127 | 0.4251 | 0.516 |
|  | visit | 6954.3 | 2318.09 | 3 | 190.99 | 69.1221 | <0.001 |
|  | drug | 220 | 110.01 | 2 | 127 | 3.2802 | 0.041 |
|  | visit:drug | 328.2 | 82.06 | 4 | 256 | 2.4468 | 0.047 |
| Change of Dizziness Frequency | age | 4.482 | 4.482 | 1 | 127 | 2.1407 | 0.146 |
|  | visit | 313.118 | 78.279 | 4 | 254.31 | 37.3892 | <0.001 |
|  | drug | 1.379 | 0.689 | 2 | 127 | 0.3292 | 0.720 |
|  | visit:drug | 2.849 | 0.475 | 6 | 384 | 0.2268 | 0.968 |
| Change of visual analogue scale for dizziness | age | 0.8 | 0.797 | 1 | 127 | 0.2205 | 0.639 |
|  | visit | 607.8 | 202.601 | 3 | 190.99 | 56.0623 | <0.001 |
|  | drug | 7.67 | 3.834 | 2 | 127 | 1.0609 | 0.349 |
|  | visit:drug | 41.63 | 10.408 | 4 | 256 | 2.8801 | 0.023 |
| Change of Dizziness Handicap Inventory scores | age | 0 | 0 | 1 | 127 | 0.0003 | 0.985 |
|  | visit | 19022.1 | 6340.7 | 3 | 190.99 | 44.5644 | <0.001 |
|  | drug | 62 | 31 | 2 | 127 | 0.2178 | 0.805 |
|  | visit:drug | 239.6 | 59.9 | 4 | 256 | 0.421 | 0.793 |
| Change of UCLA Dizziness Questionnaire | age | 1.53 | 1.53 | 1 | 127 | 0.1756 | 0.676 |
|  | visit | 1752 | 584 | 3 | 190.99 | 67.0723 | <0.001 |
|  | drug | 6.29 | 3.15 | 2 | 127 | 0.3612 | 0.698 |
|  | visit:drug | 30.17 | 7.54 | 4 | 256 | 0.8662 | 0.485 |
