# Supplementary figures and images for: Therapeutic efficacy of nimodipine and topiramate on migraine and vestibular migraine; A prospective multicenter open-label study
Source: PLoS One. 2026 Mar 19;21(3):e0344948. doi: 10.1371/journal.pone.0344948 (PMC13001945; doi:10.1371/journal.pone.0344948)

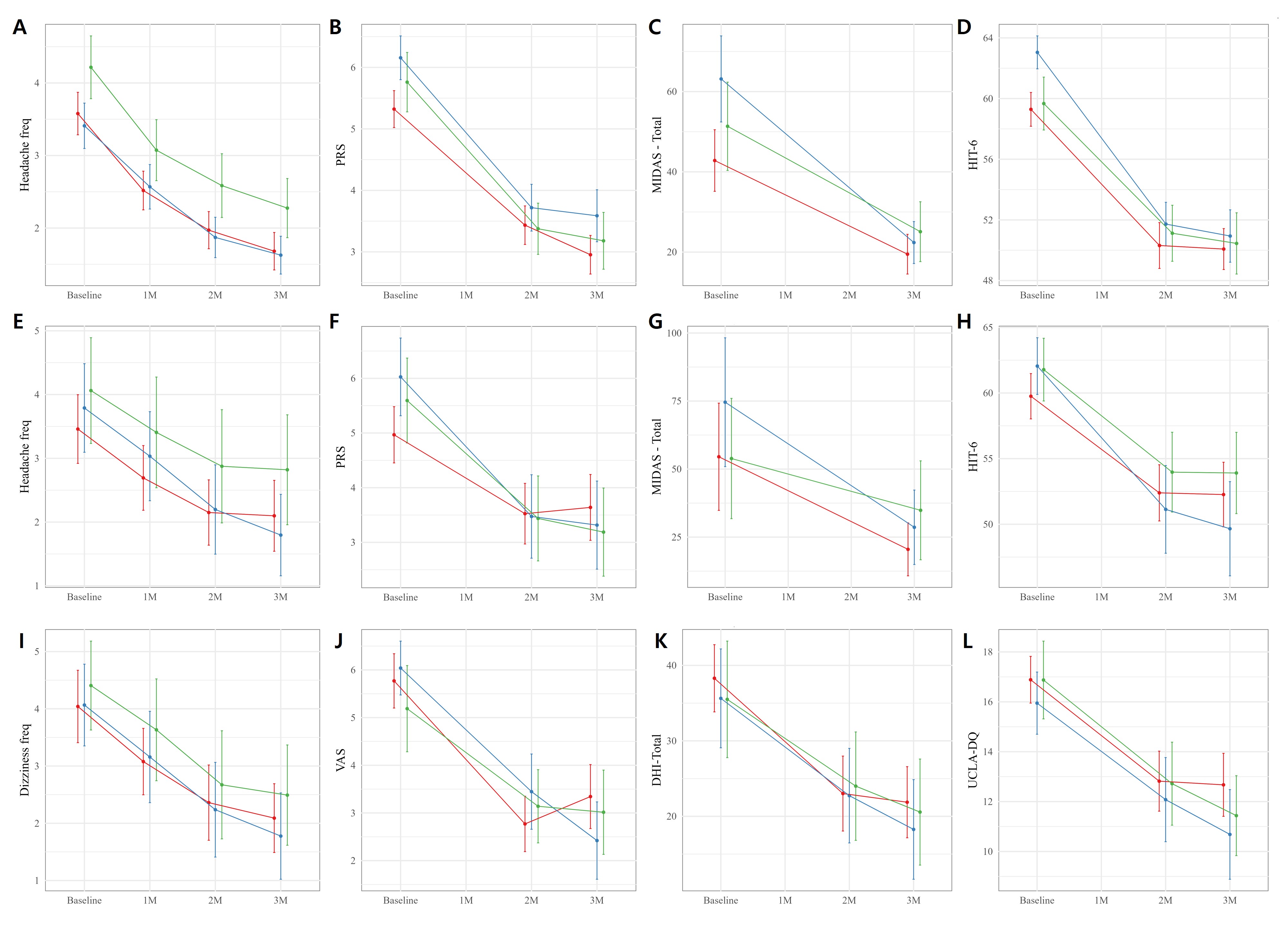

Supplement: S1 Fig — The headache days per week (A), and the scores of Wong-Baker pain rating scale (PRS, B), Migraine Disability Assessment (MIDAS-Total, C), and Headache Impact Test-6 (HIT-6, D) decreased significantly with medications in all treatment groups. Patients with vestibular migraine also showed improvements in, the headache frequency (E) and intensity (PRS, F), MIDAS-Total (G), HIT-6 (H), dizziness days per week (I), visual analogue scale for dizziness intensity (VAS, J), Dizziness Handicap Inventory (DHI, K), and UCLA Dizziness Questionnaire (UCLA-DQ, L) scores with 3 months of medication (Dots represent the mean, and bars indicate the 95% CI. Red = nimodipine, Blue = topiramate, Green = combination group). (PNG) [file pone.0344948.s006.jpg]

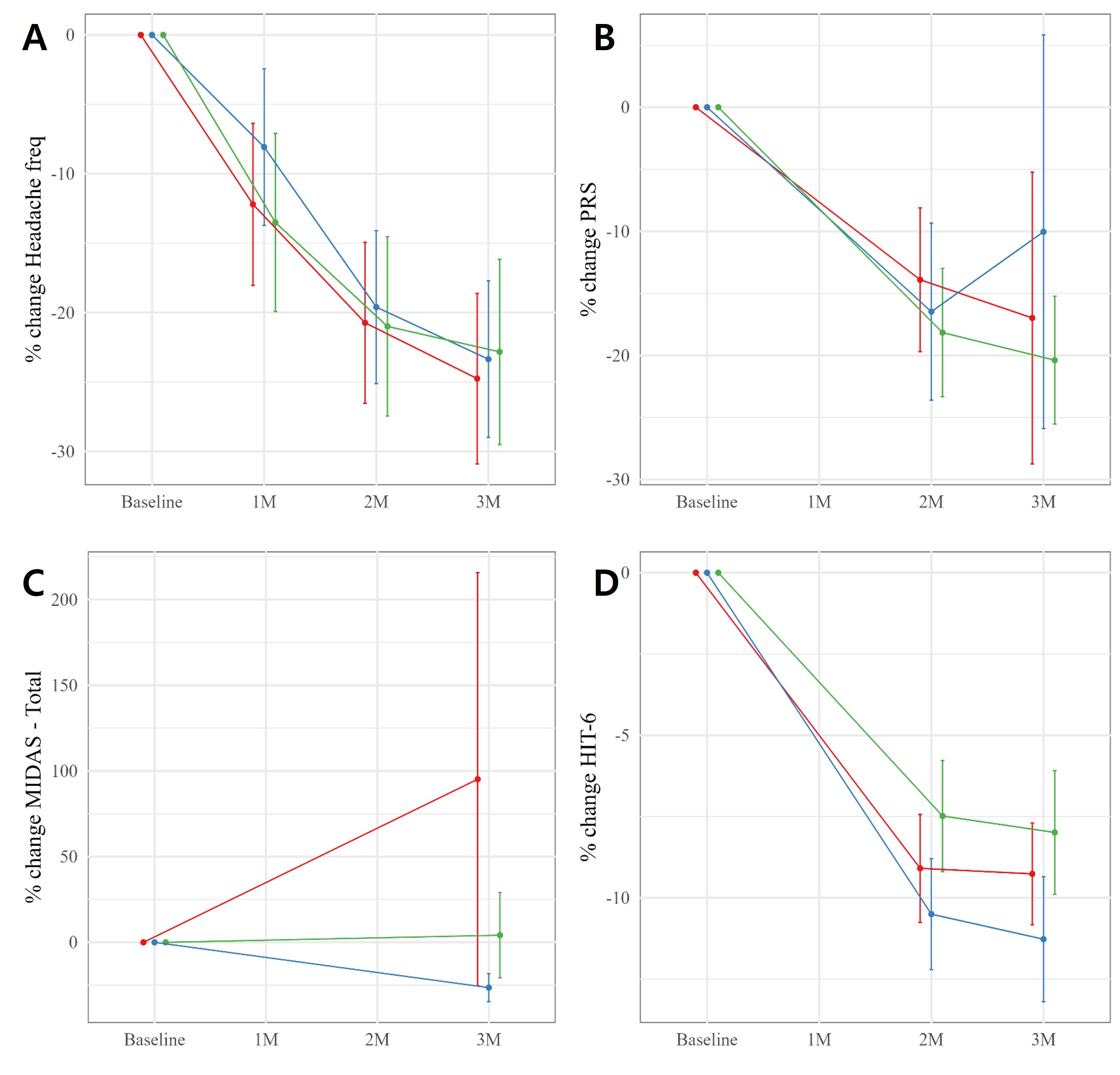

Supplement: S2 Fig — Headache frequency (A) and Wong-Baker Pain Rating Scale (PRS, B) scores significantly decreased over the three-month period in all groups (both p < 0.001), with no significant differences between groups (p = 0.870 and 0.727, respectively). In contrast, the Migraine Disability Assessment (MIDAS-total, C) score did not show a significant percent change from baseline (p = 0.604), nor were there significant inter-group differences (p = 0.139). Headache Impact Test-6 (HIT-6, D) scores decreased significantly in all groups (p < 0.001), with a significant difference observed among the three groups (p = 0.042). (Dots represent the mean and bars indicate the 95% CI. Red = nimodipine, Blue = topiramate, Green = combination group). (PNG) [file pone.0344948.s007.jpg]

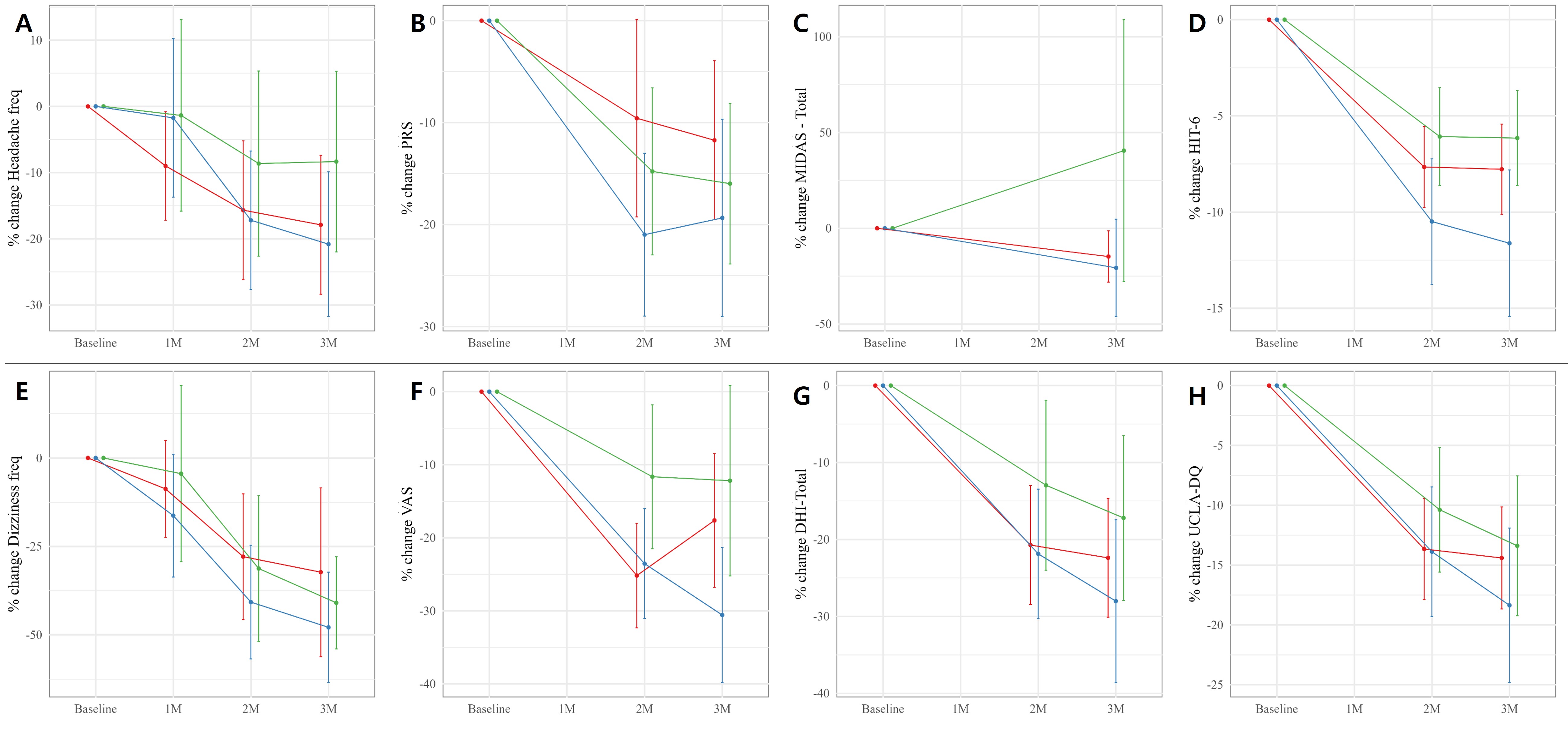

Supplement: S3 Fig — For headache-related measures (A–D), significant percent reductions from baseline were observed in headache frequency (A), Wong-Baker Pain Rating Scale (PRS, B), and Headache Impact Test-6 (HIT-6, D) scores (all p < 0.001). However, no significant percent change was observed for the MIDAS-total score (C; p = 0.931). No significant inter-group differences were found for any of these measures (% change in headache frequency: p = 0.499; PRS: p = 0.238; MIDAS-total: p = 0.053; HIT-6: p = 0.050). For dizziness-related measures (E–H) including percent change of dizziness frequency (E), visual analogue scale for dizziness intensity (VAS, F), Dizziness Handicap Inventory (DHI-Total, G), and UCLA Dizziness Questionnaire (UCLA-DQ, H), all groups showed significant reductions over three months (all p < 0.001), with no significant inter-group differences (% change in dizziness frequency: p = 0.543; VAS: p = 0.064; DHI-Total: p = 0.373; UCLA-DQ: p = 0.501). (Dots represent the mean and bars indicate the 95% CI. Red = nimodipine, Blue = topiramate, Green = combination group). (PNG) [file pone.0344948.s008.jpg]
